# ESCRT-II controls retinal axon growth by regulating DCC receptor levels and local protein synthesis

**DOI:** 10.1098/rsob.150218

**Published:** 2016-04-20

**Authors:** Filip A. Konopacki, Hovy Ho-Wai Wong, Asha Dwivedy, Anaïs Bellon, Michael D. Blower, Christine E. Holt

**Affiliations:** 1 Department of Physiology Development Neuroscience, University of Cambridge, Downing Street, Cambridge CB2 3DY, UK; 2 Department of Molecular Biology, Harvard Medical School, Simches Research Center, Boston, MA 02114, USA

**Keywords:** endocytosis, ESCRT, DCC, Netrin-1, axon guidance, protein synthesis

## Abstract

Endocytosis and local protein synthesis (LPS) act coordinately to mediate the chemotropic responses of axons, but the link between these two processes is poorly understood. The endosomal sorting complex required for transport (ESCRT) is a key regulator of cargo sorting in the endocytic pathway, and here we have investigated the role of ESCRT-II, a critical ESCRT component, in *Xenopus* retinal ganglion cell (RGC) axons. We show that ESCRT-II is present in RGC axonal growth cones (GCs) where it co-localizes with endocytic vesicle GTPases and, unexpectedly, with the Netrin-1 receptor, deleted in colorectal cancer (DCC). ESCRT-II knockdown (KD) decreases endocytosis and, strikingly, reduces DCC in GCs and leads to axon growth and guidance defects. ESCRT-II-depleted axons fail to turn in response to a Netrin-1 gradient *in vitro* and many axons fail to exit the eye *in vivo*. These defects, similar to Netrin-1/DCC loss-of-function phenotypes, can be rescued in whole (*in vitro*) or in part (*in vivo*) by expressing DCC. In addition, ESCRT-II KD impairs LPS in GCs and live imaging reveals that ESCRT-II transports mRNAs in axons. Collectively, our results show that the ESCRT-II-mediated endocytic pathway regulates both DCC and LPS in the axonal compartment and suggest that ESCRT-II aids gradient sensing in GCs by coupling endocytosis to LPS.

## 1. Introduction

During development, neurons send out axons that navigate long distances to their synaptic targets. Growth cones (GCs), located at the tips of growing axons, respond directionally to cues in their environment and are essential for this navigation. Extrinsic cues bind and activate receptors in GCs and stimulate endocytosis [1–5]. The guidance receptors deleted in colorectal cancer (DCC) and neuropilin, for example, are rapidly endocytosed upon binding Netrin-1 and Sema3A, respectively [3–6]. Endocytosis is required for the collapse response of GCs to repulsive cues [4,7] and polarized endocytosis underlies the steering responses of GCs to a cue gradient [8,9]. Gradient-elicited GC steering similarly involves a polarized pattern of de novo protein synthesis (PS) [10,11] and GC adaptation, a key feature of gradient sensing, relies on the coupled action of endocytosis and local protein synthesis (LPS) [5] suggesting that these two processes are closely linked. Indeed, mRNA transport is directly linked to endosomes in fungal hyphae where it assists with precise subcellular targeting of locally synthesized proteins [12].

Activated receptor complexes [13,14] enter the endocytic pathway and are sorted into specific vesicles from where they can continue to signal [15–18]. Correct sorting and transport of endocytosed receptors are necessary for their function in GC motility and guidance [19]. Different classes of vesicles, named early, late and recycling endosomes, carry cargoes destined for either recycling or degradation. The endosomal sorting complex required for transport (ESCRT) machinery controls the passage of endocytosed cargo between different types of endosomes. It consists mostly of vacuolar protein sorting (Vps) proteins and multiple associated factors organized into complexes named ESCRT-0, ESCRT-I, ESCRT-II and ESCRT-III [20]. The ESCRT system was originally identified for its involvement in endosomal sorting of ubiquitylated proteins and multivesicular endosome biogenesis [21]. However, ESCRT components are now known to regulate many additional cellular functions, including receptor signalling, polarity, migration, cytokinesis, viral budding, autophagy, exosome secretion, miRNA activity and mRNA transport [22–26].

While the ESCRT system commonly works together as a coordinate set of proteins [27], individual ESCRT components can function independently. Of particular interest, ESCRT-II can act as an RNA binding protein [28], and endosomes have been implicated in mRNA transport and local translation [29–31]. Cue-induced LPS in GCs is known to play a role in axon navigation [32], and guidance cue receptors involved in triggering axonal PS such as tropomyosin receptor kinase B (TrkB), Neuropilin-1 or DCC undergo ligand-induced endocytosis [5,6], linking them to the endosomal pathway. Regulation of endosomes by ESCRT plays a role in axonal branching [33,34] and pruning [35] but it is not yet known whether ESCRT proteins have a role in GC guidance. Therefore, we have investigated if ESCRT-II could play such a role. Our results show that ESCRT-II co-localizes with DCC and endosomes in GCs and provide evidence that ESCRT-II is needed for cue-induced LPS and axon guidance.

## 2. Material and methods

### 2.1. Embryos

*Xenopus laevis* embryos of either sex were obtained by *in vitro* fertilization, raised in 0.1× modified Barth's saline (MBS) at 14–18°C and staged according to the tables of Nieuwkoop and Faber (1994).

### 2.2. Constructs, morpholinos and antibodies

#### 2.2.1. Constructs

*Xenopus laevis* ESCRT-II subunits EGFP-Vps22, Vps25 and Vps36, mouse DCC and gap-GFP were cloned into pCS2 vector. GeneMachine Kit (Ambion) was used to make mRNA.

#### 2.2.2. Morpholinos

Antisense morpholino oligonucleotides (MOs) either non-modified or conjugated to carboxyfluorescein were purchased from GeneTools. *Xenopus laevis* Vps25 splice blocking MOs: 5′-GCTGTGCCCACCACACCGCTCACGT-3′ and 5′-CAACTTTCCTGGAACACAGTATGTT-3′. Control MO: 5′-CCTCTTACCTCAGTTACAATTTATA 3′.

#### 2.2.3. Antibodies

Rabbit anti-*Xenopus laevis* ESCRT-II polyclonal antibody was made in the Blower laboratory; other antibodies were obtained as follows: mouse anti-acetylated *α*-tubulin (Sigma cat. no. T6793), mouse anti-*α*-tubulin (Sigma cat. no. T6074), mouse anti-Rab4 (BD Biosciences cat. no. 610888), mouse anti-Rab5 (BD Biosciences cat. no. 610281), mouse anti-Rab7 (Sigma cat. no. R8779), mouse anti-Rab11 (BD Biosciences cat. no. 610656), rabbit anti-β-tubulin (Abcam cat. no. ab6046), mouse anti-DCC intracellular domain (BD Biosciences cat. no. 554223), goat anti-DCC extracellular domain (R&D Systems cat. no. AF844), rabbit anti-RPL5 (Proteintech Europe cat. no. 15430-1-AP).

### 2.4. Cell cultures

Eye primordia were dissected from stage 24 *X. laevis* embryos and plated on 50 mm glass-bottom dishes (MatTek) coated with 10 mg ml^−1^ poly-l-lysine and 10 mg ml^−1^ laminin (both from Sigma). Explants were cultured at 20°C in 60% Leibowitz's L15 medium (Gibco) containing 5% penicillin/streptomycin/fungizone (Sigma cat. no. P4458) and 50 µg ml^−1^ gentamycin (GE Healthcare) for 16–18 h.

### 2.5. Immunostaining

Axons growing from *Xenopus* eyes cultured in glass-bottom dishes were fixed with 2% paraformaldehyde (Pfa)/7.5% sucrose in 1× phosphate-buffered saline (PBS) for 20 min at room temperature (RT), washed with PBS, permeabilized with 0.1% Triton X-100 in PBS for 5 min at RT, blocked with 5% goat serum in PBS for 30 min at RT, then incubated with the primary antibody for 1 h at RT followed by a fluorochrome-conjugated secondary for 30 min at RT. Where appropriate, Phalloidin-Alexa Fluor 488 (an F-actin stain; 1 : 100, Invitrogen/Molecular Probes) was added together with the second antibody. GCs were mounted in Fluorosave (Calbiochem), coverslipped and imaged on a Nikon Eclipse TE2000-U inverted microscope fitted with an Hamamatsu C4742-80-12AG camera. Co-localization images were taken on a spinning disk microscope (UltraVIEW Vox system (PerkinElmer) mounted on an Olympus IX81 inverted microscope) equipped with an EM-CCD camera (C9100-50, Hamamatsu).

#### 2.5.1. Surface/total DCC staining

Axons cultured in glass-bottom dishes were fixed with 2% Pfa/7.5% sucrose in 1× PBS for 5 min on ice. After four quick washes with ice-cold PBS, the axons were incubated with goat anti-DCC extracellular domain antibody (R&D Systems cat. no. AF844) at 1 : 20 in PBS/0.1% BSA for 30 min on ice and washed 4× 2 min with ice-cold PBS. Axons were then fixed again with 2% Pfa/7.5% sucrose in 1× PBS for 20 min at RT, washed with PBS, permeabilized with 0.1% Triton X-100 in PBS for 5 min at RT and incubated with mouse anti-DCC intracellular domain antibody (BD Biosciences cat. no. 554223) for for 1 h at RT, followed by a mix of donkey secondary antibodies (anti-goat Alexa Fluor 568 and anti-mouse Alexa Fluor 647) for 30 min at RT. GCs were mounted in Fluorosave (Calbiochem), coverslipped and imaged on a Nikon Eclipse TE2000-U inverted microscope fitted with a Hamamatsu C4742-80-12AG camera.

### 2.6. Western blot

Stage 37–38 *Xenopus* embryos were anaesthetized in 0.4 mg ml^−1^ tricaine methanesulfonate (MS222) in 1× MBS and their eyes were dissected, homogenized and lysed in RIPA buffer (Sigma cat. no. R0278) containing cOmplete Mini EDTA-free protease inhibitor cocktail (Roche cat. no. 11 836 170 001) and spun on a bench-top centrifuge at 4°C to clear the lysates. The protein supernatants were resolved on 12% or 4–20% gradient Mini-Protean gels (Bio-Rad) and transferred to nitrocellulose membrane (Bio-Rad). The membrane was blocked for 30 min in 5% skimmed milk in Tris-buffered saline–Tween 20 (TBS-T: 150 mM NaCl, 50 mM Tris–HCl, pH 8.0, 0.05% Tween) at RT and incubated with primary antibodies: rabbit anti-ESCRT-II 1 : 1000 or mouse anti-DCC (BD Transduction labs) 1 : 1000 or mouse anti-*α*-tubulin (Sigma cat. no. T6074) 1 : 5000 in 5% milk/TBS-T for 2 h at RT, followed by either horseradish peroxidase-conjugated anti-rabbit or anti-mouse secondary antibody (both Abcam, 1 : 5000) for 1 h at RT. Bands were detected on GE Healthcare Hyperfilm ECL films using an ECL Plus detection kit (Amersham) and X-ray developer.

### 2.7. Electroporation

Eye-targeted electroporation was performed on stage 26–28 embryos as previously described [36]. Embryos anaesthetized with 0.4 mg ml^−1^ MS222 in 1× MBS were placed yolk-up in the longitudinal channel of a cross-shaped Sylgard chamber, so that the eyes faced the homemade platinum electrodes. A solution of 1 mg ml^−1^ DNA or 1 mM fluorescein-tagged MO was microinjected into the lumen of the eye vesicle and a 1 Hz series of eight pulses (18 V and 50 ms long) was delivered by a square wave pulse generator (TSS20 OVODYNE Electroporator, Intracel). Embryos were returned to 0.1× MBS and grown at 18°C until reaching stage 41. Embryos were either imaged live (see §2.8) or fixed in 4% Pfa in PBS overnight at 4°C, embedded in OCT compound and cryosectioned to 12 µm thick sections mounted on microscope slides. Sections were stained with DAPI and imaged on a Nikon Eclipse 80i upright microscope.

### 2.8. *In vivo* ventral brain preparation

Stage 41 embryos were lightly anaesthetized in 1× MBS with 0.4 mg ml^−1^ of MS222. The ventral surface of the brain was exposed by carefully slicing through from the position directly dorsal to the cement gland to the posterior hindbrain. The exposed brains were then bathed in 1× MBS with 0.1 mg ml^−1^ of MS222 and mounted in ventral view in order to visualize the whole optic path, and imaged on a Nikon Eclipse 80i upright microscope.

### 2.9. Microinjections

The procedure was performed as described previously [37]. Briefly, jelly coats of the *in vitro* fertilized embryos were removed with 2% cysteine (Sigma) solution in 1× MBS. MOs (12 ng per blastomere) and mRNAs were diluted in RNase-free water and microinjected into both dorsal animal blastomeres (a volume of 5 nl blastomere^−1^) at the four-cell stage in a solution of 4% Ficoll (Sigma) in 1× MBS.

### 2.10. *In vitro* axon outgrowth assay

Time-lapse recordings were performed on explanted whole *Xenopus* eye primordia from stage 24 cultured in 50 mm glass-bottom dishes (MatTek) for 16–18 h. Phase contrast images were taken every 5 min for a total of 2 h (see figure 3 for details) using 20× on a Nikon Eclipse TE2000-U inverted microscope fitted with a motorized XY stage (Prior Scientific, Cambridge, UK) and an automatic shutter. Axons from four to five explants per dish were imaged at the same time. The same axons were traced before and after adding Netrin-1. The average speed of growth was calculated in Microsoft Excel from the total distance covered by the GC in periods before and after Netrin-1 using ImageJ (FIJI) plugin Manual Tracking.

### 2.11. Growth cone turning assay

Retinal explants from stage 24 *Xenopus* eye primordia were prepared as described and plated on poly-l-lysine-coated 50 mm glass-bottom dishes (MatTek). Retinal explants were cultured for 18 h at 20°C before turning assays. Netrin-1 gradients were produced by pulsatile ejection of 10 µg ml^−1^ mouse recombinant Netrin-1 (R&D Systems cat. no. 1009-N1) solution in culture media from a glass micropipette using a pressure application system (Picospritzer, General Valve). The pipette tip was placed 100 µm from the GC at a 45° angle from the direction of growth, resulting in an approximately 10^3^-fold dilution of Netrin-1 at the GC. Phase-contrast images were acquired every 5 min for 1 h. Turning angle was defined as the angle between the original direction of growth and the direction after 1 h. Only GCs that extended 5 µm over 1 h were included in the analysis. Unlike the other *in vitro* experiments in this paper, for turning assays laminin coating was not used as it is known to interfere with attractive Netrin-1 signalling [38].

### 2.12. Proximity ligation assay

This was performed according to the manufacturer's instructions (Olink Bioscience) with modifications [39]. Briefly, eye explant cultures grown in glass-bottom dishes were fixed, permeabilized and blocked exactly as for immunostaining. The pair of primary antibodies, either mouse anti-DCC intracellular domain (1 : 100, BD Biosciences) and rabbit anti-ESCRT-II (1 : 200) or the same anti-DCC and rabbit anti-RPL5 (1 : 100, Proteintech Europe cat. no. 15430-1-AP), were applied and incubated overnight at 4°C. The dishes were washed 4× 5 min in PBS/0.002% Triton X-100 followed by application of anti-rabbit (+) and anti-mouse (−) probes for 1 h at 37°C, ligase for 30 min at 37°C and the polymerase mix with green fluorescence for 100 min at 37°C. The samples were mounted in Duolink mounting media and imaged on the same day.

### 2.13. FM4-64 dye incorporation

Experiments were performed on RGC axons growing from whole *Xenopus* eye primordia from stage 24 embryos cultured in 50 mm glass-bottom dishes (MatTek) for 16–18 h. A stock of 2 mM aqueous solution of fixable FM4-64FX (Molecular Probes/Life Technologies) was vortexed, filtered through a 0.22 µm syringe filter and diluted 1 : 200 in 60% L15 culture media. The working solution was applied to cells for 90 s at RT. Dishes were subsequently put on ice and washed 3× with ice-cold culture media before being fixed in 2% Pfa/7.5% sucrose in PBS for 20 min at RT. Fluorescent images were acquired on a Nikon TE2000-U microscope at 60× magnification. Total FM dye fluorescent intensity was measured and background subtracted in ImageJ (FIJI).

### 2.14. l-Azidohomoalanine incorporation assay

Stage 24 *Xenopus* eye explant cultures were incubated in methionine-free 60% L15 media for 6 h prior to the experiments. l-Azidohomoalanine (AHA; Life Technologies cat. no. C10102) 200 µM concentration was added for 1 h, then washed with embryo media followed by PBS. Explants were fixed with 2% Pfa/7.5% sucrose in PBS for 20 min at RT, washed 3× with 3% BSA/PBS and permeabilized with 0.1% Triton X-100 in PBS, and the Click reaction was performed with Click-iT Alexa Fluor 488 detection reagent, according to manufacturer's instructions (Life Technologies). Culture dishes were mounted in Fluorosave and imaged.

### 2.15. Puromycin labelling assay

*Xenopus* eye explants (either non-stimulated (i.e. time 0 min) or Netrin-1 (300 ng ml^−1^) stimulated for 25 or 40 min; see Results and figure 7) were incubated with 10 µg ml^−1^ puromycin (Sigma cat. no. p8833) in culture media for 10 min (the final 10 min of Netrin-1 stimulation) at RT, washed 3× with ice-cold culture media, fixed with 2% Pfa/7.5% sucrose in PBS for 20 min at RT, washed in PBS/1 µg ml^−1^ saponin, permeabilized in 10 µg ml^−1^ saponin/PBS for 5 min at RT, blocked in 5% goat serum/1 µg ml^−1^ saponin in PBS for 1 h and incubated with anti-puromycin antibody directly conjugated to Alexa Fluor 488 (Millipore/Molecular Probes cat. no. MABE343-AF488; diluted 1 : 250) overnight at 4°C in blocking solution, washed 5× 5 min in PBS/1 µg ml^−1^ saponin, mounted in Fluorosave (Calbiochem) and imaged.

### 2.16. Live imaging of ESCRT-II and mRNA

*Xenopus* embryos were co-injected with mRNAs encoding Vps25 (400 pg blastomere^−1^), EGFP-Vps22 and Vps36 (200 pg blastomere^−1^ each) and Cy3-labelled β-actin mRNA (500 pg blastomere^−1^) at the four-cell stage and eye cultures in glass-bottom dishes were prepared as described. Live movies of EGFP-ESCRT-II and Cy3-β-actin mRNA in axons were recorded using a spinning disc confocal microscope (UltraVIEW Vox system (PerkinElmer mounted on Olympus IX81 inverted microscope) equipped with an EM-CCD camera (C9100-50, Hamamatsu), controlled by Volocity software set to maximum sample protection; single plane frames were acquired every 2 s (alternating between 488 and 543 laser lines) for up to 5 min. The movies were post-processed using both Volocity and ImageJ software to suppress noise.

### 2.17. Statistical analysis

Statistical analyses were performed using GraphPad Prism v. 5 software. For each experimental group, D'Agostino and Pearson omnibus normality test was run, followed by either Anova + Bonferroni/Student's *t*-test/uncorrected Fisher's LSD (for normally distributed samples) or Kruskal–Wallis + Dunnis/Mann–Whitney test.

## 3. Results

### 3.1. ESCRT-II localizes to endosomal compartments in axonal growth cones

**Figure 1.**
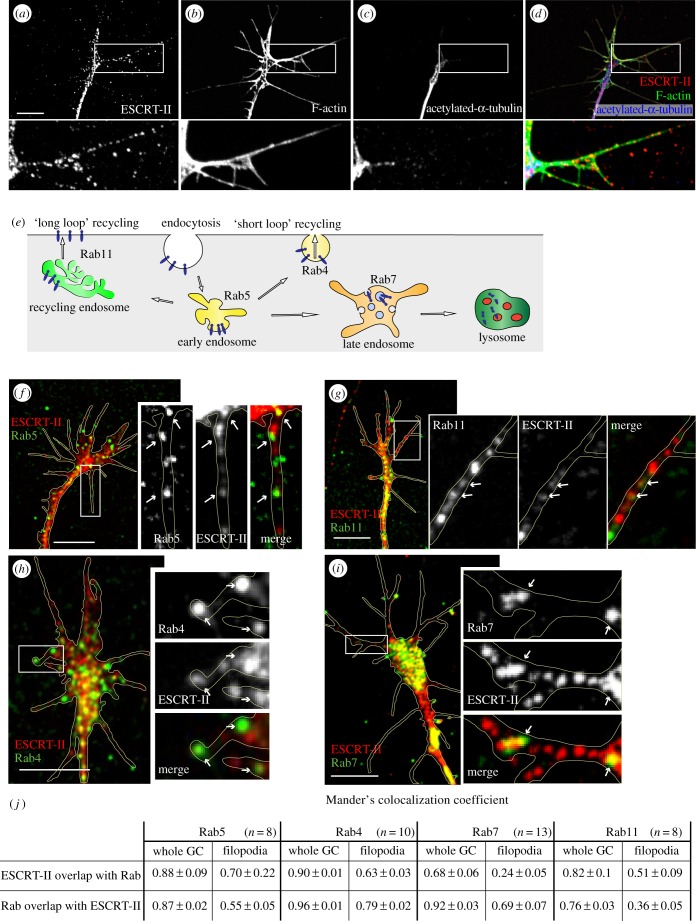
ESCRT-II co-localizes with early endosomal vesicles in RGC growth cones. (*a–d*) ESCRT-II immunoreactivity in GCs (*a*) co-labelled with F-actin (*b*; strongly labelling GC periphery including filopodia) and acetylated *α*-tubulin (*c*; predominantly staining axon shaft and GC central domain). Panel (*d*) shows a compound image of (*a–c*). Note ESCRT-II-positive granules in GC filopodia (insets *a–d*). (*e*) Cartoon depicts the involvement of individual Rabs with distinct elements of the endocytic pathway. (*f–i*) Co-localization of ESCRT-II with endosomal markers Rab5 (*f*), Rab11 (*g*), Rab4 (*h*) and Rab7 (*i*) in RGC GCs. Arrows point to spots where the signals visibly co-localize. GC outline indicated with yellow line. (*j*) Table shows average Manders' co-localization coefficients of Rabs and ESCRT-II in whole GC and filopodia. Scale bars, 10 µm.

To determine the role of ESCRT-II in axon guidance, we first examined its localization in the GC. Immunostaining of cultured RGCs with a specific anti-*Xenopus* ESCRT-II antibody revealed positive punctate staining in the axons, as well as in the central and the peripheral domains of GCs (figure 1*a–d*). ESCRT-II-positive puncta were especially evident along F-actin-rich (phalloidin-positive) filopodia [40], where they often resembled a ‘string of beads' (figure 1*a,b,d*). Stable microtubules, detected by anti-acetylated tubulin, typically localize to the GC central domain (figure 1*c*).

The localization and function of ESCRT components overlap with those of the Rab family of small GTPases [41,42]. Rab5 is associated with early endosomes, Rab4 labels ‘short loop’ recycling endosomes, Rab11 associates with ‘long loop’ recycling endosomes and Rab7 marks late endosomes which send the cargo for degradation in lysosomes [43] (figure 1*e*). We therefore assessed the levels of co-localization of these Rabs with ESCRT-II. The markers of early/recycling endosomes (Rab5 and Rab4) as well as late and ‘long loop’ recycling endosomes (Rab7 and Rab11) were found to co-localize with ESCRT-II (figure 1*f–j*). This suggests that ESCRT-II is associated with both the early and late part of the endosomal pathway, including recycling endosomes, in GCs.

### 3.2. ESCRT-II depletion reduces endocytosis

**Figure 2.**
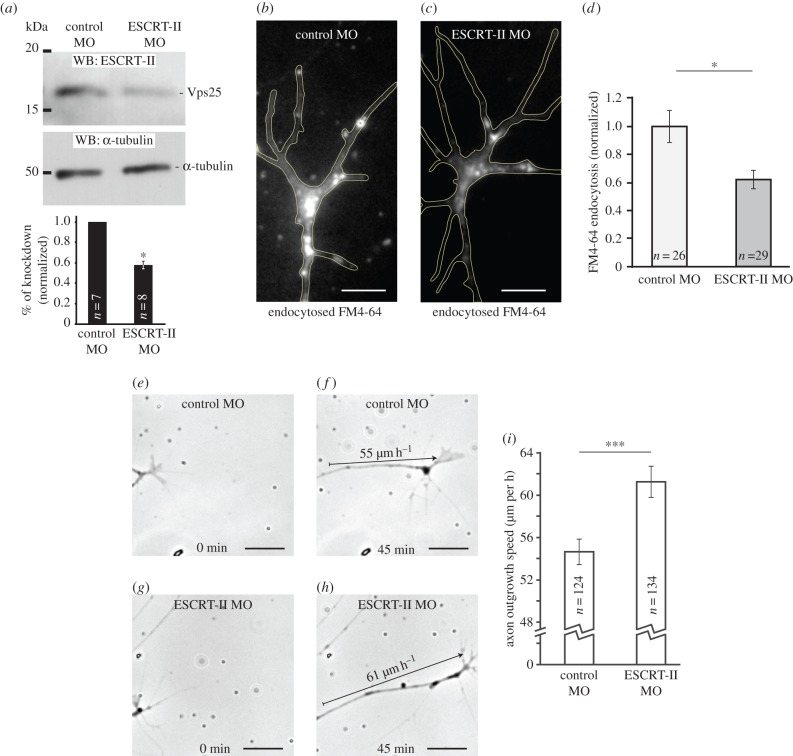
ESCRT-II knockdown reduces growth cone endocytosis and accelerates axon growth *in vitro*. (*a*) Verification of the MO-induced ESCRT-II knockdown. The morpholino targets the Vps25 subunit of ESCRT-II. Western blots were done in whole eye extracts. Graph shows the mean reduction of Vps25 band intensity in ESCRT-II MO-injected embryos normalized to control. (*b–d*) Balance of endo/exocytosis measured by the levels of FM4-64 dye loaded over 90 s into control MO (*b*) or ESCRT-II MO (*c*) GCs. GC outline indicated with yellow line. (*d*) Graph shows the average FM4-64 fluorescence in GCs normalized to control MO-injected axons. (*e–i*) *In vitro* RGC axon outgrowth assay. The growth of axons from embryos injected with control MO (*e,f*) or ESCRT-II MO (*g,h*) measured over 45 min; arrows in (*f)* and (*h*) indicate how far the axons extended. (*i*) Quantification of axon growth speed *in vitro*. *p ≤ 0.05, ***p ≤ 0.0001, Student's *t*-test. Scale bars, 5 µm (*b,c*), 20 µm (*e–h*).

Given the importance of membrane dynamics in the GC, we next investigated whether ESCRT-II is required for GC endocytosis using a loss-of-function approach. Because ESCRT function is essential for embryonic development [44], we chose a KD approach. We targeted the Vps25 subunit with a specific splice-blocking antisense morpholino (MO) and achieved a 42 ± 4.3% knockdown as measured by western blot analysis of stage 33/34 eye extracts (figure 2*a*).

To test whether ESCRT-II regulates endocytosis in RGC GCs, we exposed control- and ESCRT-II KD GCs to the styryl dye FM4-64FX [2,45] for 90 s, washed out the dye, and fixed the samples. Control GCs exhibited a robust FM4-64 signal, whereas in ESCRT-II-depleted GCs the signal was significantly reduced (figure 2*b–d*). This decrease of FM4-64 signal indicates that ESCRT-II knockdown impairs endocytosis. Alternatively, given the highly dynamic nature of GC endosomes and recycling turnover [45], it is possible that this decrease reflects an increase in the recycling rate of endocytosed vesicles and their reinsertion back into plasma membrane.

### 3.3. Axons grow abnormally when ESCRT-II is depleted

The speed of axon extension is, in part, controlled by a balanced rate of insertion and removal of lipid membrane through exocytosis and endocytosis, respectively [46–50]. When membrane insertion exceeds removal, axons extend and, therefore, a reduction in endocytosis would be predicted to increase the rate of axon extension. In line with this, measurements of basal axon extension rates *in vitro* showed that ESCRT-II-depleted GCs advance faster than controls (average 61.3 ± 1.5 µm h^−1^ versus 54.6 ± 1.22 µm h^−1^; figure 2*e–i*).

**Figure 3.**
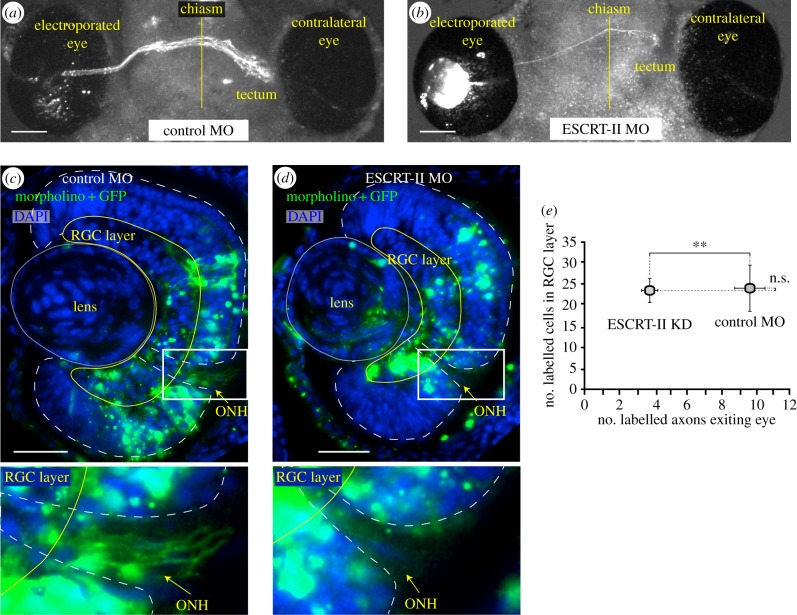
ESCRT-II knockdown impairs axon exit from the eye. (*a,b*) Images of *in vivo* ventral preparation of *Xenopus* embryos electroporated with GFP + control MO (*a*) or GFP + ESCRT-II MO (*b*). Note very few axons exiting the eye and coursing to optic tectum in (*b*). A vertical yellow line indicates the midline. (*c,d*) Sections of embryos' eyes electroporated with GFP + either a control MO (*c*; *n* = 6) or ESCRT-II MO (*d*; *n* = 7) stained with DAPI (blue) and GFP (green). (*e*) Quantification of (*c*) and (*d*). Graph shows the average number of labelled cells (vertical axis) and the corresponding number of axons in the optic path (horizontal axis) in both conditions. **p ≤ 0.001, Student's *t*-test. ONH, optic nerve head. Scale bars, 100 µm (*a,b*), 50 µm (*c,d*).

We next asked whether ESCRT-II is required for normal axon growth *in vivo*. We used electroporation to target the MOs (together with a reporter gap-GFP plasmid) directly into retinal cells at stage 26 when RGCs are newly born. We found that GFP-labelled axons exiting the eye, crossing the chiasm and entering the optic tract were markedly sparser in ESCRT-II MO-electroporated brains (less than five axons) compared with the control MO-electroporated ones (more than 10 axons; figure 3*a,b*). Even though the eye size was unchanged in the ESCRT-II MO electroporated embryos, this difference could potentially reflect fewer ESCRT-II-MO-positive cells in the retina owing to increased cell death. To address this possibility, we sectioned the electroporated eyes and counted the number of axons and the number of electroporated (MO- and GFP-positive) cells in the DAPI-stained RGC layer. On average, there were 9.7 ± 0.88 labelled axons exiting the control MO-electroporated eye, whereas the mean number of axons exiting the ESCRT-II MO-electroporated eyes was significantly reduced to 3.7 ± 0.47. The average number of MO-positive cell bodies in the RGC layer, however, was similar in both conditions (23.8 ± 5.64 control MO and 23.1 ± 2.75 ESCRT-II MO; figure 3*c–e*), suggesting that the decrease of RGC axons exiting the eye was not a secondary effect owing to RGC cell death. Collectively, the results indicate that ESCRT-II-depleted RGCs show axon growth defects *in vivo* as well as *in vitro*.

### 3.4. ESCRT-II regulates the response of retinal ganglion cell axons to Netrin-1

**Figure 4.**
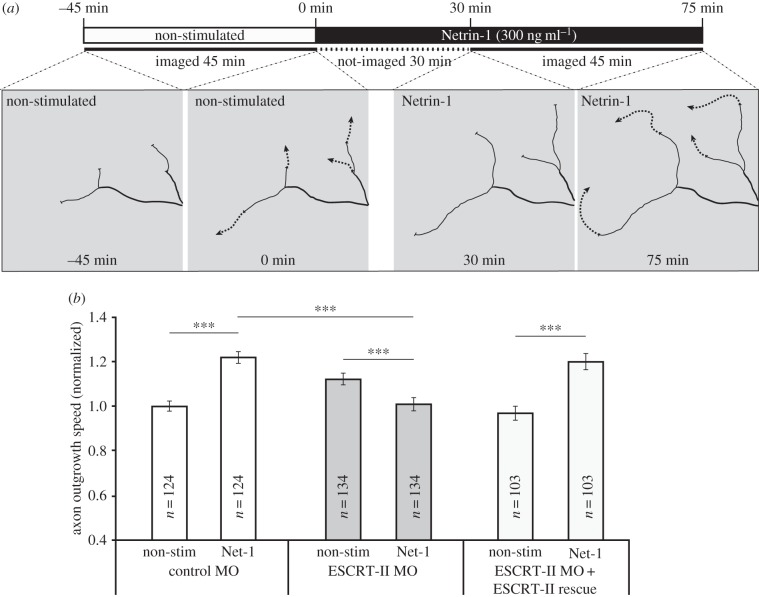
Impaired Netrin-1 responsiveness in ESCRT-II-depleted growth cones. (*a*) *In vitro* RGC axon outgrowth assay. The experimental layout is shown on the top. The growth of axons from embryos injected with control MO, ESCRT-II MO and ESCRT-II MO + ESCRT-II mRNAs was measured from time −45 min to 0 min (without Netrin-1) and subsequently from 30 min to 75 min (with Netrin-1). Drawings show representative examples of quantified axons. (*b*) Quantification of (*a*); ***p ≤ 0.0001, paired (except for comparison of bars 2 and 4) Student's *t*-test.

One of the key ligands known to guide axons out of the eye is Netrin-1, which is expressed exclusively at the optic nerve head [51–53]. We therefore asked if Netrin-1 responsiveness was affected in ESCRT-II-depleted neurons. Addition of Netrin-1 globally to cultured retinal axons stimulates an increase in the rate of axon extension by more than 40% [53]. We tested if ESCRT-II morphants retained their sensitivity to Netrin-1. Addition of 300 ng ml^−1^ Netrin-1 to the culture medium increased the speed of axon growth of control MO-injected axons by 21.9 ± 2.5% but it slightly decreased the speed of growth of ESCRT-II-depleted axons. This effect was rescued by ESCRT-II expression, which restored axon speed to control Netrin-1 stimulated levels (figure 4*a,b*). This indicates that ESCRT-II is involved in regulating Netrin-1 sensitivity.

### 3.5. ESCRT-II co-localizes and regulates DCC levels

**Figure 5.**
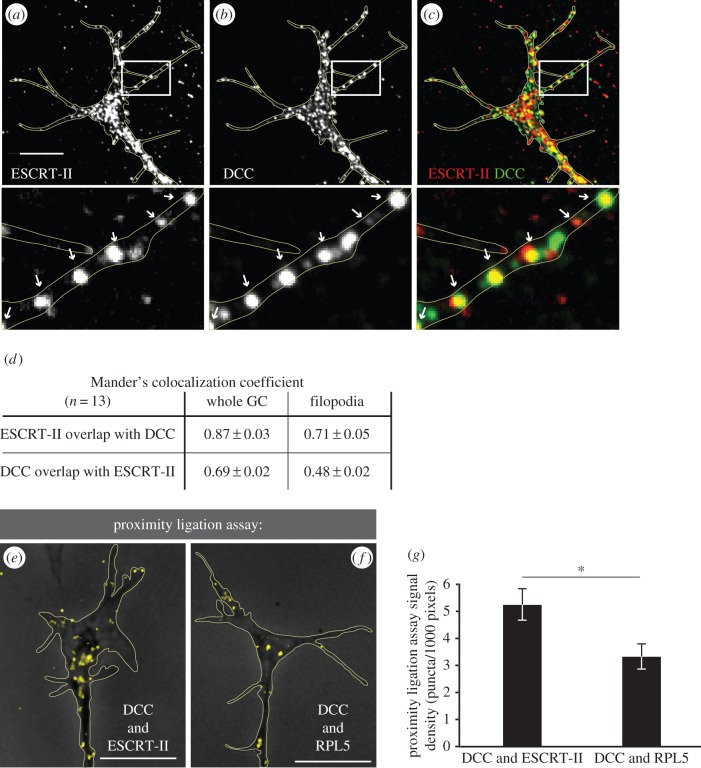
ESCRT-II co-localizes with DCC in growth cones. (*a–c*) Co-localization of ESCRT-II (*a*; red in *c*) and DCC (*b*; green in *c*) immunofluorescent signals in *Xenopus* RGC GCs. The signal overlap is especially visible in filopodia (indicated with arrows on insets below). (*d*) Table shows Manders' co-localization of DCC and ESCRT-II in whole GC and filopodia. (*e–g*) Proximity ligation assay confirming the close localization of ESCRT-II and DCC in RGC GCs (*e*). Yellow dots denote the sites where both probes interact. The known interaction of DCC with the large ribosomal subunit protein RPL5 [55] was used as a positive control (*f*). GCs are outlined with yellow lines. Graph (*g*) shows the quantification of the number of PLA puncta per unit area. *p ≤ 0.05, Student's *t*-test. Scale bars, 10 µm.

Previous findings showing that ligand-induced endocytosis of Netrin-1's receptor, DCC, is necessary to mediate Netrin-1 signalling in GCs [5] prompted us to investigate a potential link between ESCRT-II and DCC. Co-immunolabelling for DCC and ESCRT-II showed a high degree of overlap between the two signals in the GC central domain and filopodia (figure 5*a–d*) that was not altered by 10 min Netrin-1 (data not shown). To further validate DCC–ESCRT-II co-localization, we used the proximity ligation assay (PLA) [39,54], which detects when two proteins are 40 nm or less apart and, therefore, potentially interacting. GCs showed a strong DCC–ESCRT-II PLA signal (figure 5*e,g*). Interestingly, when compared with the PLA signal between DCC and one of its known direct-binding partners, RPL5 [55], the PLA signal of ESCRT-II–DCC was significantly more abundant (figure 5*e–g*). This suggests the possibility that ESCRT-II and DCC interact directly in the GC.

**Figure 6.**
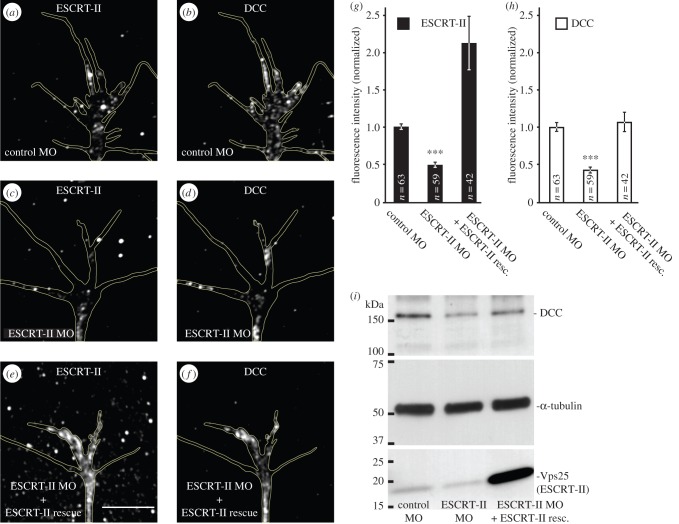
ESCRT-II regulates the levels of DCC receptor in growth cones. (*a–h*) ESCRT-II knockdown leads to decreased DCC levels in GCs. (*a–f*) Representative examples of GCs from embryos injected with control MO (*a,b*), ESCRT-II MO (*c,d*) and ESCRT-II MO + ESCRT-II mRNAs (*e,f*), stained for ESCRT-II (*a,c,e*) and DCC (*b,d,f*). (*g,h*) Graphs showing the normalized signal intensities of ESCRT-II (*g*; black bars) and DCC (*h*; white bars). ***p ≤ 0.0001 compared with control, Students' *t*-test. (*i*) A representative western blot from eye extracts indicating that the decrease in DCC levels shown in (*b,d*) is global. GCs are outlined with yellow lines. Scale bars, 10 µm.

Remarkably, we found that when ESCRT-II was reduced by 50.5 ± 4.3% with ESCRT-II MO (figure 6*a,c,g*), DCC was correspondingly decreased (51.5 ± 6.5%; figure 6*b,d,h*). This decrease was rescued by overexpression of ESCRT-II restoring the levels of DCC to 106.6 ± 13.3% of control (figure 6*e–h*). Western blot analysis on whole eyes confirmed these ESCRT-II-associated changes in DCC levels (figure 6*i*). These results indicate that ESCRT-II regulates DCC levels.

**Figure 7.**
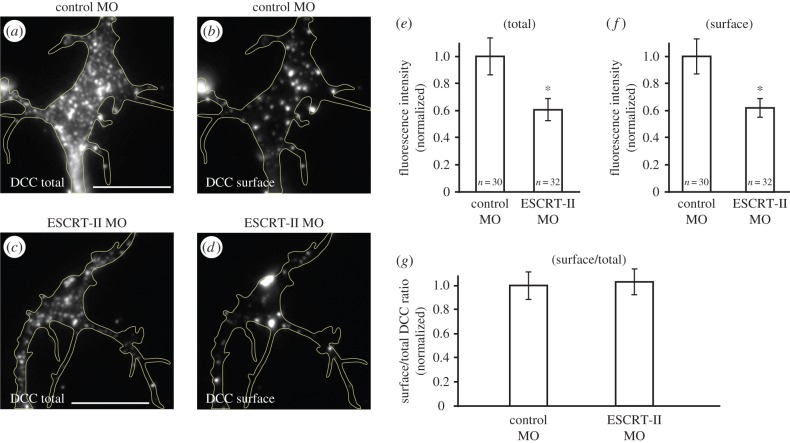
ESCRT-II regulates surface levels of DCC in growth cones. (*a–d*) Immunostaining for total (*a,c*) and surface (*b,d*) DCC receptor in control (*a,b*) and ESCRT-II-depleted (*c,d*) GCs. For clarity, the signal intensities in (*b,d*) are increased by 30% compared with (*a,c*). GCs are outlined with yellow lines. (*e–g*) Graphs showing quantification of total (*e*) and surface (*f*) DCC levels and surface to total DCC ratios (*g*), normalized to the respective controls. *p ≤ 0.05 Mann–Whitney test. Scale bars, 10 µm.

The impaired endocytosis (or faster recycling) in ESCRT-II morphant axons (figure 2*b–d*) indicates that DCC could be specifically inserted or removed from the GC plasma membrane. To test this possibility, we assessed the surface fraction of the DCC receptor using an antibody against the extracellular domain in non-permeabilized conditions. We found that in ESCRT-II-depleted GCs, the total DCC levels were reduced by 39.4 ± 8.3% (figure 7*a,c,e*), whereas the corresponding surface fraction was reduced by 38.1 ± 7.6% (figure 7*b,d,f*), compared with control GCs. The surface-to-total DCC ratios were almost identical in control and ESCRT-II-deficient GCs (figure 7*g*), indicating that the loss of total DCC is accompanied by a proportional loss of the DCC at the plasma membrane, which is consistent with the deficient response to Netrin-1 (figure 4).

### 3.6. DCC rescues ESCRT-II deficiency

**Figure 8.**
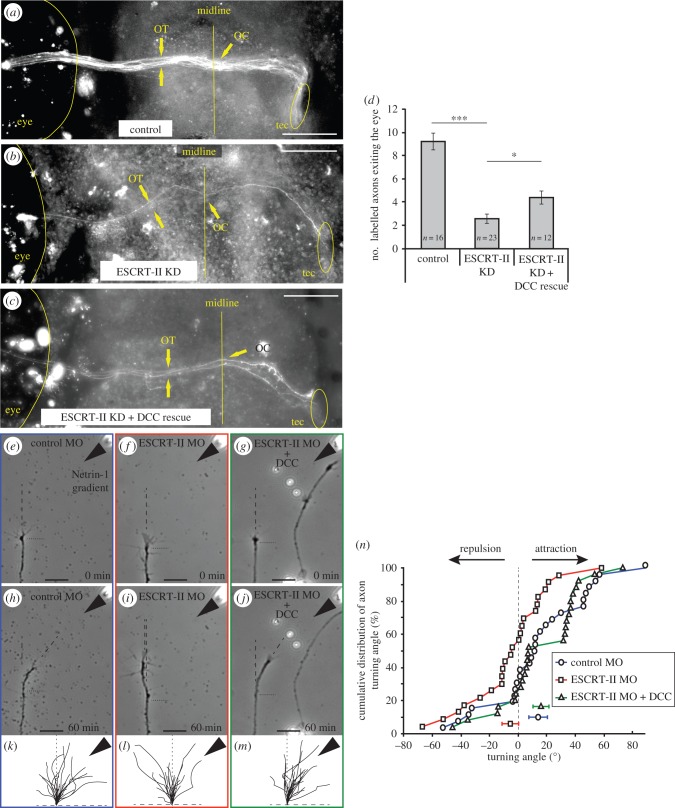
DCC rescues ESCRT-II knockdown phenotypes. (*a–d*) *In vivo* ventral view of the *Xenopus* optic path in stage 41 embryos whose right eye had been electroporated with control MO (*a*), ESCRT-II MO (*b*) and ESCRT-II MO + DCC mRNA (*c*). The numbers of axons exiting the eye and navigating in the optic pathway were counted and the quantification is shown in (*d*). OT, optic tract; OC, optic chiasm; tec, optic tectum. (*e–o*) *In vitro* turning assay. (*e–j*) Representative examples of RGC axons from embryos injected with control MO (*e,h*), ESCRT-II MO (*f,i*) and ESCRT-II MO + DCC mRNA (*g,j*) before (*e–g*) and after (*h–j*) being subjected to a Netrin-1 gradient ejected from a pipette (indicated with black arrowheads) set at 45° angle from the direction of growth. Growth measurement start point is indicated with horizontal black dotted line; dashed lines show the measured directions of growth at time 0 min and 45 min. (*k–m*) Traces of control (*k*), ESCRT-II MO (*l*) and ESCRT-II MO + DCC mRNA (*m*) axons growing for 1 h while exposed to Netrin-1 gradient (black arrowheads). (*n*) Cumulative distribution plot showing the turning angles of all measured axons. *p ≤ 0.05, ANOVA + uncorrected LSD Fisher's test. Scale bars, 20 µm.

The finding that both total and surface DCC decrease with ESCRT-II depletion raised the possibility that DCC loss underlies the ESCRT-II phenotype. If this is correct, we reasoned that restoring DCC levels should rescue the ESCRT-II phenotype. To address this, we electroporated a DCC-expression plasmid together with ESCRT-II MO and a gap-GFP reporter into stage 26 *Xenopus* eyes and counted the number of fluorescent axons exiting the eyes and navigating along the pathway to the optic tectum at stage 41 (figure 8*a–d*). ESCRT-II morphants (figure 8*b*) had significantly fewer axons in the optic pathway than control embryos (2.50 ± 0.4 axons versus 9.25 ± 0.73; figure 8*a,d*). Co-electroporation of ESCRT-II MO with the DCC-expressing plasmid resulted in an increase of the number of labelled axons exiting the eye (4.40 ± 0.58; figure 8*c,d*), indicating that DCC overexpression is sufficient to at least partially rescue the *in vivo* phenotypes in ESCRT-II knockdown.

We therefore hypothesized that DCC expression could restore sensitivity to Netrin-1 in ESCRT-II-depleted GCs. To test this, we performed a turning assay, where growing axons *in vitro* are exposed to a gradient of guidance cue [56,57]. In agreement with previous studies [53], control MO-injected RGC axons showed a strong attractive turning response to a Netrin-1 gradient (mean turning angle + 13.78 ± 6.49°, figure 8*e,h,k,n*). As expected, ESCRT-II-depleted axons exhibited no directional response to a Netrin-1 gradient (mean turning angle of −5.59 ± 5.76°, figure 8*f,i,l,n*). However, re-expression of DCC in ESCRT-II-depleted axons restored their responsiveness to Netrin-1 (mean turning angle of + 15.77 ± 5.56°, figure 8*g,j,m,n*). This suggests that the regulation of DCC by ESCRT-II is needed for the Netrin-1 gradient-sensing turning mechanism.

### 3.7. ESCRT-II knockdown reduces local protein synthesis

**Figure 9.**
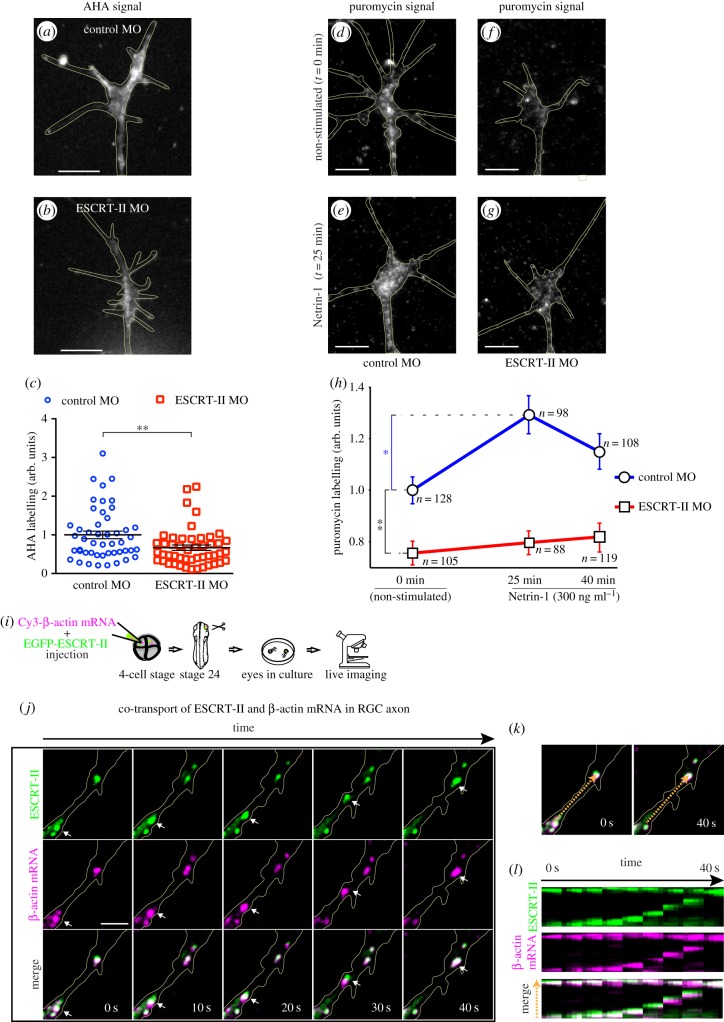
ESCRT-II KD decreases local protein synthesis and ESCRT-II-mRNA positive granules are trafficked along axons. (*a–h*) De novo protein synthesis in *Xenopus* RGC GCs *in vitro*. (*a–c*) AHA incorporation over 1 hour into non-stimulated RGC GCs. (*a,b*) Representative images of control (*a*) and ESCRT-II KD (*b*) GCs. (*c*) Graph showing the comparison of the two conditions. **p ≤ 0.01, Mann–Whitney test. (*d–h*) Puromycin labelling in GCs in response to bath application of Netrin-1 (300 ng ml^−1^) for 0, 25 and 40 min. (*d–g*) Representative images of control (*d,e*) and ESCRT-II KD (*f,g*) GCs fixed at time 0 (no stimulation: *d,f*) and 25 min of Netrin-1 stimulation (*e,g*). In each case, puromycin was added 10 min before fixation. (*h*) Graph represents measured fluorescence levels, indicative of puromycin labelling (see Material and methods). Blue trace, control MO; red trace, ESCRT-II knockdown. Data normalized to non-stimulated control MO. *p ≤ 0.05, **p ≤ 0.01, Kruskal–Wallis test with Dunnis post hoc. GCs outlined in yellow. Scale bars, 5 µm. (*i–j*) ESCRT-II co-transport with β-actin mRNA in RGC axon. (*i*) Cartoon showing the experimental design. (*j*) Time-lapse (12 fpm) imaging of an RGC axon *in vitro* (outlined in yellow) expressing EGFP-ESCRT-II (green) and Cy3-β-actin mRNA (magenta). Co-localization is visible as white on merge images. Arrows point to an example of an ESCRT-II-positive granule moving together with Cy3-labelled β-actin mRNA. Scale bar, 5 µm. (*k,l*) A kymograph of the area marked with orange broken arrow in the merge images (*k*), showing the movement of one ESCRT-II and β-actin mRNA-positive granule (bottom to top of the three kymographs in *l*), while another one remains stationary (at the top of each kymograph in *l*).

DCC activation by Netrin-1 triggers LPS in GCs [58], and the local translation of β-actin mRNA has been shown to mediate GC turning *in vitro* [10,11]. Given that ESCRT-II has been identified as an RNA-binding protein [28], we asked if ESCRT-II depletion affects PS in the GC. We assayed the de novo PS using the AHA incorporation assay [59]. ESCRT-II knockdown resulted in significantly reduced baseline levels of PS in GCs (67 ± 7% of control, figure 9*a–c*). The 1-h period of incubation required for AHA labelling meant that this method could not be used to assess the rapid Netrin-1-induced PS [58]. Instead, we took advantage of the aminonucleoside antibiotic puromycin, which at low concentrations can be used to label nascent proteins without blocking translation [60–63], and we quantified the signal using a fluorescent anti-puromycin antibody [63]. In line with previous studies [58], Netrin-1 stimulation caused a significant increase in puromycin signal in control GCs, indicative of increased LPS (figure 9*d,e,h*). By contrast, in ESCRT-II-depleted GCs, puromycin staining did not show any increase with Netrin-1 stimulation (figure 9*f–h*). Consistent with the AHA labelling experiment, ESCRT-II morphants exhibited a much lower level of basal PS (0 min; figure 9*h*). These results show that ESCRT-II abolishes Netrin-1-stimulated PS in GCs consistent with a loss of DCC signalling. In addition, ESCRT-II KD substantially lowers basal (unstimulated) levels of de novo PS in GCs.

Because the basal level of translation is decreased in ESCRT-II KD GCs and ESCRT-II is reportedly an RNA-binding protein [28], we asked whether ESCRT-II transports mRNA. β-actin mRNA is abundant in RGC GCs [64], and is locally translated in response to Netrin-1 in RGC GCs [10]. ESCRT-II–GFP plasmid and fluorescently labelled β-actin mRNA were electroporated into retinal neurons, and time-lapse imaging was subsequently performed on axons in retinal explant cultures (figure 9*i*). We observed dynamic granules of ESCRT-II–GFP trafficking along axons in both anterograde and retrograde directions and these were commonly positive for β-actin mRNA (figure 9*j–l* and electronic supplementary material, movie S1). These results show that ESCRT-II and mRNAs co-localize and travel together in the same RNA granules and suggest that ESCRT-II may play an active role in RNA trafficking along axons in neurons.

## 4. Discussion

To date, only a few studies have investigated ESCRT machinery in developing axons and these have focused primarily on ESCRT-0 and ESCRT-III [34,35]. Here, we have characterized for the first time, to the best of our knowledge, the localization of ESCRT-II in *Xenopus* RGC GCs and have investigated the effect of ESCRT-II depletion on retinal axon growth *in vitro* and *in vivo*. Briefly, our results show that (i) ESCRT-II associates with the endosomal pathway in GCs; (ii) ESCRT-II KD impairs axon exit from the eye *in vivo* and abolishes Netrin-1-guided responses *in vitro*; (iii) ESCRT-II depletion causes a loss of DCC in GCs and DCC expression rescues defects in axon growth; (iv) ESCRT-II depletion lowers the basal rate of PS in the GC and abolishes Netrin-1-induced PS increase; and (v) ESCRT-II co-transports with mRNA in axons.

The ESCRT system is classically known to sort and package ubiquitylated cargo into the intraluminal vesicles inside late endosomes, thus forming multivesicular bodies (MVBs) [21]. Our results show that that ESCRT-II also co-localizes with early and recycling endosomes. We find that ESCRT-II KD causes a decrease in FM4-64 dye loading, indicating that ESCRT-II could have an early function in the dynamic regulation of the endocytosis and/or recycling and re-insertion of the endosomes into the plasma membrane of RGC GCs.

Indeed, recent studies link ESCRT machinery to the early/recycling part of the endosomal system. For example, Hrs (ESCRT-0) was found to associate with a subset of clathrin pits at the plasma membrane, where it sorts surface cargo even before endocytosis to facilitate downstream trafficking [65]. On the other hand, one of ESCRT-III's subunits, Ist1, was shown to interact with spastin in early endosomes, controlling the fission of recycling endosomes and the sorting of recycling cargoes away from the degradation pathway [34]. These studies, together with our results, indicate that ESCRT-II could potentially interact with other components of the ESCRT machinery to control the dynamics of endosomal recycling.

Although the canonical view holds that the multiprotein ESCRT system acts as a whole to control cargo sorting, membrane bending and fission [21], emerging evidence suggests that individual elements of the ESCRT system can act independently of each other. For example, the ESCRT-II subunit Vps25 acts independently of Hrs to decrease Notch signalling in *Drosophila* imaginal discs [66], and the ESCRT-III element Vps32 regulates exovesicle secretion via a mechanism that is distinct from MVB formation [67]. ESCRT-II has been shown to work independently of ESCRT-I and III in regulating degradation but not in the recycling of EGFR [68]. Interestingly, the reported role of ESCRT-II as an RNA binding protein in *Drosophila* oocytes [28] also did not require other ESCRTs. It is possible, therefore, that in *Xenopus* RGC growth cones, ESCRT-II also acts in a non-canonical way. Thus, even though previous studies showed that some ESCRT components, such as ESCRT-0 [65], are dispensable for endocytosis, we cannot exclude an independent role of ESCRT-II in the regulation of endocytosis in RGC growth cones. In particular, ESCRTs have previously been suggested to control surface levels of various receptors, indicating their possible involvement in endo- and/or exocytosis. For example, overexpression of the ESCRT-III component CHMP6 leads to increased removal of the transferrin receptor from the plasma membrane [69], whereas disruption of Vps25 function causes increased surface presence of active Notch [66,70]. Taken together, this indicates that ESCRT-II might act in a non-canonical as well as canonical way, to regulate the dynamics between endocytosis and recycling/exocytosis.

Our results indicate that ESCRT-II associates with endosomal vesicles that can contribute to membrane removal. The balance between endocytosis and exocytosis in growth cones is a key regulator of axon growth: excess exocytosis permits extension, whereas excess endocytosis enables retraction [46–50]. Our finding that ESCRT-II KD leads to accelerated growth of RGC axons *in vitro* could arise from a shift in the balance of endocytosis/exocytosis with the insertion of new membrane by exocytosis exceeding membrane removal by endocytosis, thereby fuelling increased extension. Interestingly, it appears that GC morphology and advance critically depend on the correct localization of endosomes. For example, recent evidence shows that specifically directing Rab11 endosomes into or out of the GC leads to axon growth enhancement or suppression, respectively [71]. In future studies, it will be of interest to assess the exact role of ESCRT-II in endosomal trafficking and membrane flow. The accelerated growth is unlikely to be due to the loss of DCC receptor in ESCRT-II morphants, because DCC activation increases axon growth [72].

The finding that DCC rescues the axon guidance defects in ESCRT-II-depleted axons both *in vivo* and *in vitro* indicates that the loss of DCC receptor, primarily, underlies the axon guidance phenotypes. ESCRT-II morphants exhibited reduced retinal projections with fewer axons exiting the eye and crossing the diencephalon. This defect is similar to Netrin-1 hypomorphs and DCC knockout mice [51] and probably arises owing to a failure of axons to turn into the optic nerve head, a key choice point known to require Netrin-1–DCC signalling [51,53]. In addition, ESCRT-II-depleted axons, like DCC-compromised axons [53], are not able to turn in a Netrin-1 gradient.

Although DCC fully rescued the turning defect *in vitro*, the rescue was only partial *in vivo*, so alternative and/or additional hypotheses should be considered. For instance, the reduced number of axons exiting the ESCRT-II morphant eyes could be due, at least partially, to disrupted axogenesis. The robust growth of ESCRT-II-depleted axons observed *in vitro*, however, argues against this possibility. Another hypothesis is that ESCRT-II KD affects other guidance mechanisms that ultimately cause RGC axons to miss the entrance to the optic nerve head. For example, mice lacking Slit-1 and Slit-2 also exhibit axon misrouting at the optic nerve head [73], and the Slit-2 response is dependent on both endocytosis and new PS [74]. Additionally, other guidance systems for axon growth involving EphB and L1 within the retina [75,76] are regulated by endocytosis [7,77,78] and show similarities when knocked down to the ESCRT-II knockdown phenotype [75,76], suggesting that ESCRT-II might also regulate these pathways.

Our results show that ESCRT-II regulates the levels of DCC receptor in the GC, although the mechanism for this is not clear. The high degree of co-localization of ESCRT-II and DCC in GCs, confirmed by the PLA assay, suggests that they may interact directly. One possibility is that ESCRT-II binds DCC in the soma and transports it along the axon to the GC. Our finding that the levels of both surface and internal DCC in the growth cone decrease by the same amount with ESCRT-II knockdown is consistent with a global mechanism of this sort, rather than a local one. Another possibility is that the loss of axonal PS caused by ESCRT-II KD eliminates the local de novo supply of DCC. The reappearance of DCC on the GC surface after its removal through Netrin-1-induced endocytosis is sensitive to PS inhibition [5]. However, there is no direct evidence that DCC itself is axonally synthesized. DCC is rapidly endocytosed after Netrin-1-stimulation [5,79] and subsequently degraded [79]. Thus, an alternative possibility is that by associating with early/recycling endosomes, ESCRT-II could protect DCC from Netrin-1-induced degradation, although the unchanged surface/total ratio of DCC in ESCRT-II KD argues against this. It is notable that ESCRT-II morphant GCs do not respond to a Netrin-1 gradient *in vitro* despite losing only about 50% of their DCC. This suggests that ESCRT-II function may be involved in the gradient sensing mechanism, where the stoichiometric proportions of DCC to other Netrin-1 receptors (e.g. neogenin or UNC-5) could be crucial for correctly responding directionally to guidance cues. On the other hand, the Netrin-1-induced endocytosis of the remaining 50% of DCC left in ESCRT-II morphant GCs could contribute to the decrease of axon speed *in vitro*.

Our live imaging experiments provide direct evidence that ESCRT-II–GFP associates with β-actin mRNA in moving granules in retinal axons. This observation indicates that ESCRT-II may bind and transport mRNA in axons and suggests that it may play a major role in RNA localization in axons. This is consistent with results in *Drosophila* showing that ESCRT-II binds and localizes bicoid mRNA in the oocyte [28]. The mobile ESCRT-II-mRNA-labelled granules in axons are reminiscent of the endosome–mRNA granules seen in fungal hyphae [30]. These long cellular structures, somewhat analogous to axons, are known to transport mRNA on endosomes to distant subcellular locations where de novo PS takes place locally [12]. If ESCRT-II plays a major role in mRNA transport in axons, this could provide an explanation for the significant reduction in basal levels of axonal PS following ESCRT-II KD. If fewer mRNAs reach the axon, then it follows that there will be a drop in LPS. In future studies, it will be interesting to investigate whether ESCRT-II-depleted axons harbour fewer mRNAs.

In summary, our findings indicate that ESCRT-II is involved in controlling RGC axonal guidance through DCC signalling. Furthermore, the simultaneous loss of endocytosis and basal LPS suggests that ESCRT-II plays a novel role in the coordinate regulation of these two processes. Our studies raise new questions about how ESCRT-II functions in axonal GCs, and particularly whether its role in the early steps of endocytosis and endosomal sorting to the recycling pathway is the primary mechanism underlying these functions.
